# Diabetic cheiroarthropathy in uncontrolled type 2 diabetes with positive anti-nuclear antibodies: a case report from Sudan

**DOI:** 10.1097/MS9.0000000000001993

**Published:** 2024-03-21

**Authors:** Elham Abdalla, Abrar Mohamed Gamar, Ziryab Imad Taha, Mohammed Alfatih

**Affiliations:** a Department of Internal Medicine, Bahri University, Khartoum Sudan; b Faculty of Medicine, Al-Zaiem Al-Azhari University, Khartoum, Sudan/Clinical Immunology Resident, Sudan Medical Specialization Board, Khartoum, Sudan; c Rheumatology Speciality Department, Sudan Medical Specialization Board, Khartoum, Sudan; d Alzaiem Alazhari University, Khartoum, Sudan

**Keywords:** anti-nuclear antibodies, bilateral carpal tunnel syndrome, diabetes mellitus, diabetic cheiroarthropathy, Dupuytren’s contracture, prayer sign, tabletop sign

## Abstract

**Background:**

Diabetic cheiroarthropathy, also known as limited joint mobility, is one of the long-standing complications of type 2 diabetes mellitus (DM). It affects 8–50% of patients with type 1 diabetes and is also seen in type 2 diabetic patients. Consequently, it can mimic many rheumatological diseases and is often underdiagnosed. The authors present a case of a long-standing poorly controlled diabetes with diabetic cheiroarthropathy and diabetic neuropathy, along with positive ANA in the absence of any correlated autoimmune or rheumatological diseases.

**Case presentation:**

A 52-year-old female patient with poorly controlled diabetes (her last HbA1c reading was 9.5%) presented to the Rheumatology clinic with flexion deformities of the fingers. The patient has impaired vibration, two-point discrimination, and pinprick sensation in gloves and stock distribution, indicating peripheral neuropathy, entrapment neuropathy in the forms of bilateral carpal tunnel syndrome, and the diagnosis of diabetic cheiroarthropathy was made. Additionally, she has a positive prayer sign and a tabletop sign. Despite the absence of symptoms and signs of autoimmune disorders, this patient has positive anti-nuclear antibodies global (ANA positive by indirect immuno-fluorescence (IIF) 1\320 nucleolar pattern) with a negative: ANA profile, rheumatoid factor (RF) and anticyclic citrullinated peptide antibody (ACPA).

**Conclusion:**

Regular and careful hand examination should be part of clinical assessment for diabetic patients as it could be a very simple and useful screening tool for diabetic cheiroarthropathy. Physicians can use this condition as a mirror for microvascular complications of diabetes. This allows for early detection and appropriate interventions to prevent further progression of diabetes-related complications. It is also essential to consider the presence of positive ANA in diabetic cheiroarthropathy despite the absence of any rheumatological and autoimmune diseases.

## Introduction

Highlights

- Diabetic cheiroarthropathy is one of the musculoskeletal manifestations of diabetes mellitus, and it’s often linked to the duration of diabetes mellitus, poor glycemic control, and microvascular complications.
- Diabetic cheiroarthropathy is a neglected long-term complication of diabetes, causing physical limitations in performing skilled fine motor activities and emotional distress.
- Diabetic cheiroarthropathy is often underdiagnosed, and early recognition and management of diabetic cheiroarthropathy can lead to symptom improvement and potentially prevent further complications.
- Routine screening for diabetic cheiroarthropathy can be a useful tool to detect microvascular complications like diabetic neuropathy, nephropathy and retinopathy, especially in developing countries.
- Diabetic Cheiroarthropathy is reversible in some patients if detected early.
- Autoimmunity, as indicated by positive anti-nuclear antibodies, can play a role in both diabetes and associated complications.
- Clinicians should be aware of diabetic cheiroarthropathy and its connection to diabetes management and microvascular complications.

Diabetic cheiroarthropathy, also known as limited joint mobility, is one of the neglected long-term complications of type 2 diabetes mellitus (DM), which can lead to physical limitations in performing skilled fine motor activities and thereby cause emotional distress^1^. This condition is characterized by thickened skin and limited mobility of the joints in the hands and fingers, and it can be associated with complications like Dupuytren’s contracture and flexor tenosynovitis^2^. The prevalence of diabetic cheiroarthropathy varies across studies, and it affects 8–50% of patients with type 1 diabetes and is also seen in type 2 diabetic patients^3^.

Diabetic cheiroarthropathy can mimic many rheumatological diseases (i.e. rheumatoid arthritis, osteoarthritis, gout, and systemic sclerosis) and is often underdiagnosed, in contrast to micro- and macro-vascular complications^4^. Symptoms can vary from mild to severe in flexion deformity, and they usually start from the distal interphalangeal joints and progress proximally^4^.

anti-nuclear antibodies (ANA) are a class of antibodies that bind to cellular components in the nucleus, including proteins, DNA, RNA, and nucleic acid-protein complexes^5^. This test is diagnostic for connective tissue diseases, and it is associated with autoimmune diseases, idiopathic fibrosing alveolitis alveolitis-related disorders, and Raynaud’s phenomenon. Furthermore, ANA is associated with a decreased risk of hepatitis C, convulsions, fever of unknown origin and several non-autoimmune diseases^6^.

Here, we present a case of long-standing poorly controlled diabetes with diabetic cheiroarthropathy and diabetic neuropathy, along with positive ANA. This case report is in line with the CARE guidelines for case reports^7^.

## Case presentation

A 52-year-old female with a 20-year history of type 2 DM, was referred to us in the rheumatology clinic with reported numbness, pain and stiffness in both hands of one-year duration after 19 years of from the onset of type 2 DM. She denied any history of hand trauma, joint pain, swelling, sicca symptoms, or photosensitivity. She was on insulin therapy, and her glycemic control was poor, with her last (HbA1c) reading was 9.5%.

Hand examination revealed a fixed flexion deformity at the distal interphalangeal joints, accompanied by skin tightening; also, she has evidence of Duputren’s and thenar atrophy for both hands. In 2020, she was diagnosed with bilateral carpal tunnel syndrome and underwent surgical decompression for her right hand after the failure of conservative measures and local corticosteroid injection. Additionally, she is married and has not had a live baby, and she had three first-trimester miscarriages in her 30s; at that time, the cause of the miscarriages was unclear for her abortions (antiphospholipid antibodies tested twice were negative), and her miscarriages were unexplained. It was suggested that these miscarriages might be linked to her uncontrolled diabetes. Furthermore, the patient has impaired vibration, two-point discrimination, and pinprick sensation in gloves and stock distribution, indicating peripheral neuropathy.

**Figure 1:**
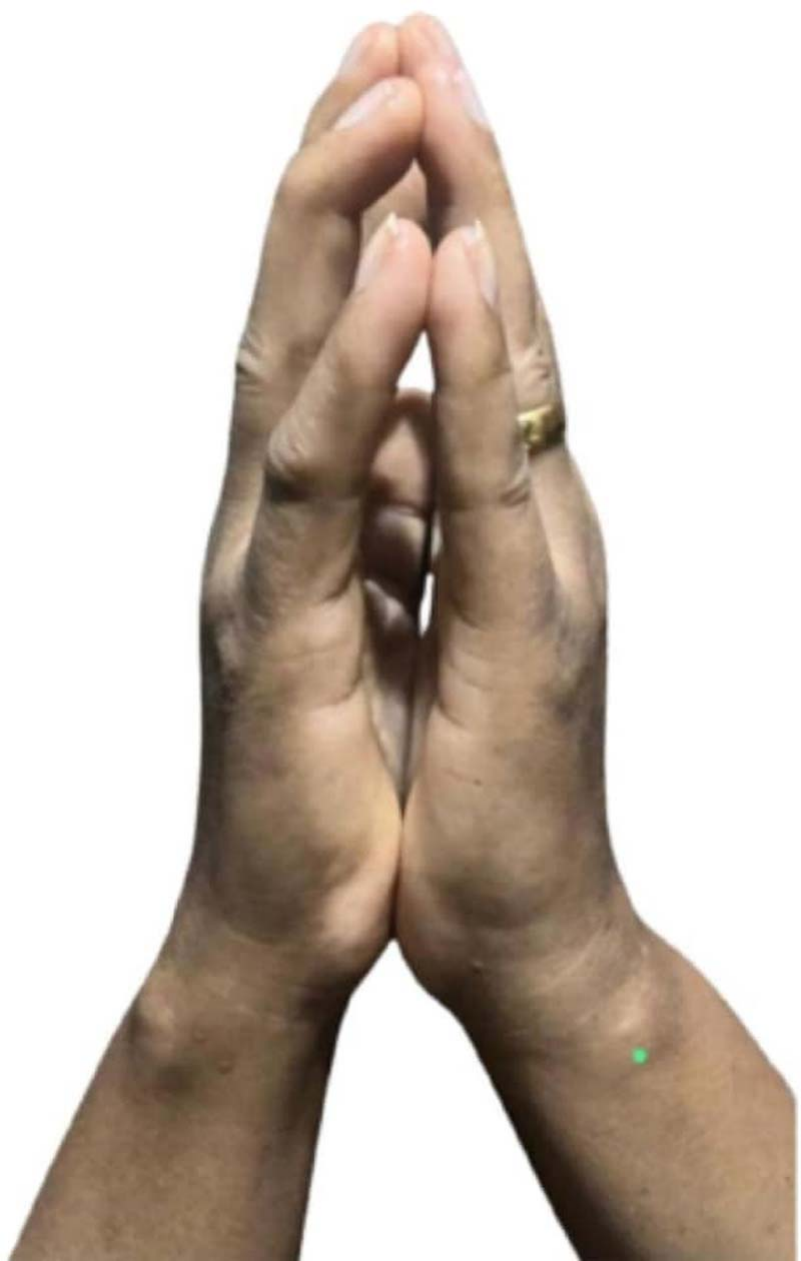
Showed positive prayer sign.

**Figure 2:**
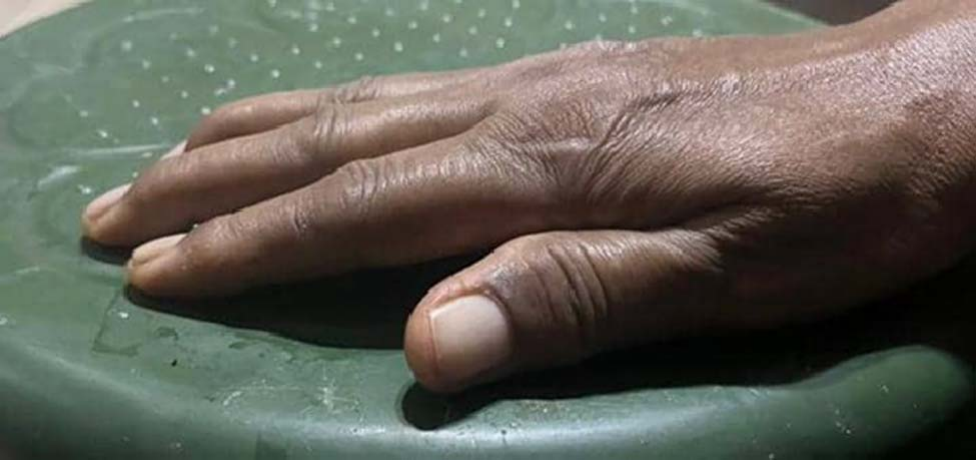
Showed a positive tabletop.

Diabetic cheiroarthropathy was diagnosed clinically based on history and positive prayer sign and tabletop sign. Furthermore, absent evidence of other underlying rheumatic disorders, particularly scleroderma and other autoimmune diseases, for example osteoarthritis. Under normal circumstances, the palmar surface of each hand can fully oppose the corresponding surface of the other hand when the hands are pressed together, resembling a prayer sign. If this action cannot be performed as seen with this patient, the sign is considered positive (Fig. 1). In addition, she was not able to lay her palms completely flat on the table at a certain spot; this indicates that there are finger flexion contractures, suggests a positive tabletop sign (Fig. 2).

### Differential diagnosis and investigations

**Figure 3:**
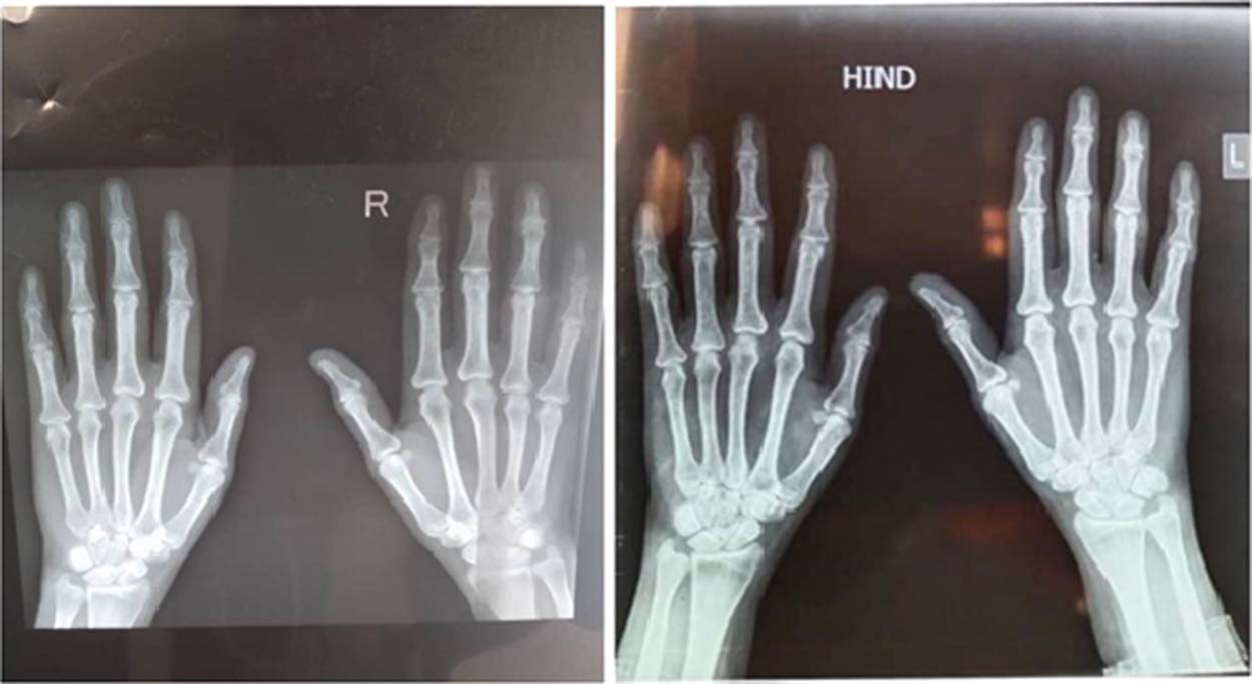
Showed normal hands X ray.

Her investigations showed a negative serology for rheumatoid factor (RF) and ACPA; Despite the negative immunological markers and ANA profile, this patient has positive ANA global (ANA by IIF 1/320 a nucleolar pattern). Other laboratory investigations, such as erythrocyte sedimentation rate, were 46 mm/hr. The liver function test was normal. Thyroid function test showed a slight increase in free T3, which was 7.43 pmol/l (reference lab value is 3.1–6.8), with normal free T4 and TSH, 11.56 pmol/l and 4.4 µIIU/ml, respectively. These thyroid laboratory findings may be due to the lab error as the patient denies any symptoms related to thyroid disease, and we can’t find any clinical interpretation for the slight increase of free T3 level. Additionally, the thyroid examination was normal. Radiographs of both hands were normal (Fig. 3).

Systemic sclerosis was ruled out owing to the absence of the Raynaud’s phenomenon. Furthermore, the tightness is not just confined to the hands. As for another differential, which is hand osteoarthritis, there were no Heberden’s nodes or Bouchard’s nodes and no radiological evidence of osteoarthritis. One of the differentials for carpal tunnel syndrome is cervical radiculopathy, especially in C6; however, she has no neck pain, and there is no limitation in her cervical mobility. Furthermore, her pain is distal to her wrists, and she has weakness in her median nerve distribution (weak thumb abductor). Spurling manoeuvre was used to rule out cervical radiculopathy. We performed this manoeuvre by turning the patient's head to one side and extending her head posteriorly, then applying downward pressure to the top of the patient. The test was negative because she did not feel pain in her arm. There was evidence of carpal tunnel syndrome, which was confirmed by positive Tinel’s and Phalen’s tests.

Flexor tenosynovitis was ruled out due to the absence of palpable crepitus. Finally, there was no evidence of trigger finger and adhesive capsulitis of the shoulder. Further assessments of the other diabetic complications revealed microalbuminuria, indicating diabetic nephropathy, while blood urea nitrogen, creatinine and electrolytes were normal. She did not have evidence of diabetic retinopathy on Fundoscopy.

### Treatment, outcome, and follow‐up

Regarding the management plan, the patient was placed on regular glucose monitoring, and the patient was advised to restrict with diet control and do regular exercises. We also educate the patient about diabetes and its complications. Additionally, we referred her for follow-up with an endocrinologist to improve her glycemic control, and then came to us in the rheumatology clinic for follow-up visits. The endocrinologist increased the dose of insulin and did serial fasting blood glucose measurements, which showed readings within a normal range after six months. Furthermore, we referred her for physiotherapy to do joint and muscle stretching exercises for the hand to reduce the pain and improve mobility. We did not give the patient a local corticosteroid injection because of its side effects and controversial results. We planned for her to visit the rheumatology clinic every 6 months to assess the outcome of treatment in terms of hand symptoms and glycemic control (to do HbA1c and compare it to previous reading) and to do a full work-up for other micro and macrovascular complications DM as well as other autoimmune diseases related to the positive ANA. Regrettably, the patient could not come to do these investigations, and we just followed up with her by telephone due to the ongoing conflict in Sudan. Regarding the outcome, our patient was placed on regular glucose monitoring, adjustment to her insulin dosages, dietary modification, and regular exercise, which led to improvement in her glycemic control. She also underwent stretching exercises, and her hand symptoms improved.

## Discussion

As the incidence and prevalence of diabetes-related complications continue to rise, it is crucial to identify musculoskeletal complications in clinical practice to enable early diagnosis^8^. Various reports have indicated that diabetic cheiroarthropathy is one of the most common musculoskeletal disorders among diabetic patients^8^. According to the study involving general practitioners, it is noteworthy that a significant percentage of them are unaware that limited joint mobility syndrome can be a complication of type 2 diabetes, and their lack of awareness regarding upper extremity disorders ranges from 59 to 73%^9^.

Antibodies have been the subject of research in diabetic patients for several years. The study involving patients with both type 1 and type 2 DM investigated autoimmunity markers and discovered that ANA antibodies were present in 24% of people with type 1 DM and 22% of those with type 2 DM. Additionally, a correlation was observed between the presence of ANA and the occurrence of diabetic polyneuropathy^10^. Another study concluded that ANA was notably present in the blood serum of patients with diabetic peripheral neuropathy, suggesting that peripheral diabetic neuropathy may have an autoimmune origin^11^.

In contrast to our findings, there is a case report that revealed that ANA was not present in the serum of patients with Diabetic cheiroarthropathy. Additionally, there was no evidence of Dupuytren contracture or carpal tunnel syndrome in their case^12^.

In this report, we present a case involving diabetic neuropathy and diabetic cheiroarthropathy with a positive ANA reading (ANA by IIF 1/320 with a nucleolar pattern). Autoimmunity serves as a shared pathogenic factor for both DM and ANA. It is also essential to consider the presence of positive ANA in healthy individuals, as they can be found in approximately 25% of the general population, typically in low titres with a dense, fine-speckled pattern. Furthermore, several studies have shown that diabetic cheiroarthropathy is directly related to the duration of diabetes mellitus and the existence of microvascular problems when compared to diabetic patients without diabetic cheiroarthropathy^13,14^.

The pathogenesis of diabetic cheiroarthropathy arises mainly from the damaging effects of hyperglycaemia and oxygen-free radicals on collagen. A genetic component has been demonstrated in some literature. An accepted hypothesis is that the joint tissue damage in diabetic patients is caused by an excess production of advanced glycation end products (AGEs) at a slow but constant rate, accumulating over time because of the increased availability of glucose in diabetic patients. AGEs form covalent cross-links in low turnover tissues, such as cartilage, bone, and tendon, via two different pathways, that is the enzymatically and the non-enzymatic glycation, which in turn change their structure and function^14^. Extensive accumulation of AGEs occurs in these tissues because the only way to degrade AGEs is when the proteins are linked to be degraded.

Tissue hypoxia is due to microvascular abnormalities and can lead to the production of oxygen-free radicals, which can then cause overproduction of growth factors and several cytokines that cause cellular damage^14^.

The Dupuytren’s contracture in diabetic patients has been reported to be 16–42%. The pathogenesis of Dupuytren’s contracture in diabetic patients has been a big debate, and some authors reported that the pathogenesis might be the same as that for cheiroarthropathy^15^. Although a strong genetics contribution with 21 dysregulated genes was demonstrated in the literature, These genes are presumed to be involved in the uncontrolled proliferation of fibroblasts and abnormal folding of α-smooth muscle actin, and moreover, these genes modulate the contractility of myofibroblasts^16^.

The treatment for diabetic cheiroarthropathy primarily involves optimizing glycemic control and providing symptomatic relief for pain management, including corticosteroid injections and stretching exercises. There have been some reports in the literature to support the idea that diabetic cheiroarthropathy is reversible in some patients if we improve glycemic control and detect it early. Recently, in animal models, novel therapies targeting AGEs have been tried but are still not recommended for clinical use in humans due to safety uncertainty and a lack of supporting evidence. These include aminoguanidine, pyridoxamine, alagebrium, and benfotiamine^17^. Surgery may be considered for individuals with persistent and severe symptoms, which can improve symptoms, although some problems may remain^14,18^. Our patient was placed on regular glucose monitoring, regular exercise, and dietary modification, which led to an improvement in her glycemic control. She also underwent physiotherapy, and her symptoms improved without the need for surgery.

The case is limited by the following facts: in practice, for any patient with bilateral carpal tunnel syndrome, it is mandatory to do cervical imaging to rule out cervical pathology, for example cervical radiculopathy. At that point, the patient was requested to do a cervical MRI and to do other investigations to rule out other autoimmune diseases related to the positive ANA. Regrettably, due to the ongoing conflict in Sudan, she could not do these investigations, and even we could not continue the follow-up physically, so we just followed up with her by telephone.

In conclusion, in developing countries, due to a lack of awareness, poor adherence to treatment and follow-up lead to poor glycemic control and an increased risk of diabetic cheiroarthropathy. Physicians should educate the patient about diabetes and its complications, which can help the patient achieve better glycemic control and reduce the risk of diabetes complications.

## Conclusion

Diabetic cheiroarthropathy is a neglected long-term complication of diabetes, which can lead to physical limitations in performing skilled fine motor activities and thereby cause emotional distress. Physicians should be aware of this condition and its associations with microvascular complications of diabetes.

Regular examination of diabetic patients for diabetic cheiroarthropathy can be a very simple and useful screening tool. Physicians can use it as a mirror for microvascular complications such as diabetic nephropathy, retinopathy, neuropathy, etc. Hence, diabetic cheiroarthropathy is an indication of poor glycemic control, and it’s the most common and long-term complication of DM. Therefore, regular and careful hand examination should be part of clinical assessment for diabetic patients, especially in developing countries, including Sudan, due to a lack of awareness and the fact that most patients have poor adherence to recommended routine screening, follow-up, and medications, which in turn leads to poor glycemic control and diabetic complications. Additionally, if the screening for microvascular complications was not performed routinely or previously, as is a very common issue in developing countries, especially in rural areas, Work-up for microvascular complications may be advised as a mandatory measure in patients with diabetic cheiroarthropathy to detect these microvascular complications at an early stage. This allows for the early detection of these complications and the implementation of appropriate interventions to prevent further progression of diabetes-related complications. Regrettably, in our case, due to the ongoing conflict in Sudan, the patient was unable to do a cervical MRI and other investigations to rule out other autoimmune diseases related to the positive ANA. Even we could not continue the follow-up physically, so we just followed up with her by telephone, and she stated that her symptoms improved despite a lack of proper follow-up and good adherence to physiotherapy due to the ongoing conflict in Sudan.

## Ethical approval

Ethical approval was not required.

## Consent

Written informed consent was obtained from the patient for publication of this case report and accompanying images.

## Sources of funding

No fund was received to this study.

## Author contribution

E.A. conceived the idea, set the study design and collected the data. E.A., A.M.G. and Z.I.T. took full detailed history, did examinations and investigations. E.A. and M.A. wrote the first draft. It was critically revised by E.A., A.M.G., Z.I.T. M.A. performed the literature review.

## Conflicts of interest disclosure

The authors report no conflict of interest.

## Research registration unique identifying number (UIN)

Not applicable.

## Guarantor

Mohammed Alfatih.

## Data availability statement

Not applicable.

## Provenance and peer review

Not commissioned, externally peer-reviewed.

## The patient perspective

The patient wasn’t aware that her symptoms were due to DM and was concerned about irreversible hand damage, even after starting treatment.
